# Supplementary material for: Unveiling the Mechanism of Phonon-Polariton Damping in α-MoO3
Source: ACS Photonics. 2024 Aug 23;11(9):3570–7. doi: 10.1021/acsphotonics.4c00485 (PMC11413844; doi:10.1021/acsphotonics.4c00485)
Supplement: Supplementary file 1 — ph4c00485_si_001.pdf [file ph4c00485_si_001.pdf]

Supporting Information

Unveiling the Mechanism of Phonon-Polariton Damping in α-MoO_3

Javier Taboada-Gutiérrez[1], Yixi Zhou[2], Ana I. F. Tresguerres-Mata[3], Christian Lanza[3], Abel Martínez-Suárez[3], Gonzalo Álvarez-Pérez[3,4], Jiahua Duan[3,4], José Ignacio Martín[3,4], María Vélez[3,4], Iván Prieto[5], Adrien Bercher[1], Jérémie Teyssier[1], Ion Errea[6,7,8], Alexey Y. Nikitin[8,9], Javier Martín-Sánchez[3,4], Alexey B. Kuzmenko[1*] and Pablo Alonso-González[3,4*]*

[*]e-mail: javiermartin@uniovi.es, Alexey.KuzMenko@unige.ch, pabloalonso@uniovi.es

[1]Department of Quantum Matter Physics, Université de Genève, 24 Quai Ernest Ansermet, CH-1211, Geneva, Switzerland

[2] Beijing Key Laboratory of Nano-Photonics and Nano-Structure (NPNS), Department of Physics, Capital Normal University, Beijing 100048, China

[3]Department of Physics, University of Oviedo, Oviedo 33006, Spain

[4]Center of Research on Nanomaterials and Nanotechnology, CINN (CSIC-Universidad de Oviedo), El Entrego 33940, Spain

[5]Institute of Science and Technology Austria, Klosterneuburg 3400, Austria

[6]Fisika Aplikatua Saila, Gipuzkoako Ingeniaritza Eskola, University of the Basque Country (UPV/EHU), Europa Plaza 1, 20018 Donostia/San Sebastián, Spain

[7]Centro de Física de Materiales (CSIC-UPV/EHU), Manuel de Lardizabal Pasealekua 5, 20018 Donostia/San Sebastián, Spain

[8]Donostia International Physics Center, Manuel de Lardizabal Pasealekua 4, 20018 Donostia/San Sebastián, Spain

[9]IKERBASQUE, Basque Foundation for Science, Bilbao, 48013 Spain

Contents

S1. Scattering-Type Scanning Near-Field Optical Microscopy (s-SNOM) and Fourier Transform Infrared Spectroscopy (FTIR)

S2. Calculated Spectra of the Dielectric Function and Reflectivity for the Normal Angle of Incidence

S3. Effect of the Finite Angle of Incidence on the Experimental Reflectivity Spectra

S4. Polaritonic Wavelength, Group Velocity and Propagation Length: Experiment vs. Theory

S5. Temperature-Dependent Study of the Polaritonic Lifetime in the Elliptical Band

S1. Scattering-Type Scanning Near-Field Optical Microscopy (s-SNOM) and Fourier Transform Infrared Spectroscopy (FTIR).

Scattering-type scanning near-field optical microscopy (s-SNOM) is a technique that allows one to excite and collect background-free highly confined polaritons with subwavelength resolution. It is based on an Atomic Force Microscope (AFM), where a metal-coated (Pt-Ir alloy) oscillating tip is illuminated with continuous wave laser sources through a parabolic mirror. The AFM tip acts thus as an optical nanoantenna, concentrating the illuminating electromagnetic field into highly localized electromagnetic near-fields at the tip apex allowing us to effectively excite polaritons in our samples. Simultaneously, it scatters towards the far-field the evanescence fields that are created at the sample surface and die off within a range of few hundreds of nanometers from the surface. Importantly, the radius of the tip apex determines the maximum resolution attainable as it establishes the volume in which the incident field is concentrated regardless of its wavelength. Furthermore, the incident light is driven through a Michelson interferometer, which in combination with the oscillation of the AFM tip enables access to background-free information of the amplitude and phase of the evanescent fields created at the sample surface. Detailed description of the system can be found elsewhere[1,2]. A sketch of the system is presented in Figure S1.

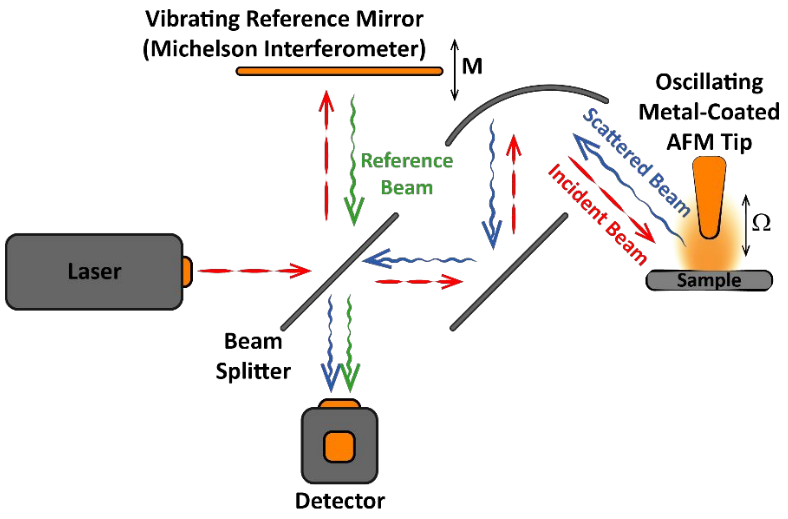

Figure S1. Principle of s-SNOM. The laser light is divided into two optical paths (sample and reference) by a beam splitter. The radiation in the sample path is focused via a parabolic mirror on the oscillating (in the so-called tapping mode) metal-coated AFM tip. These evanescent fields created by the tip are scattered back to the far-field, returning to the beam splitter by the same optical path. On the other hand, the beam splitter also directs part of the light to a vibrating

reference mirror that acts as a Michelson interferometer. The recombined reference and sample beams are finally driven to the detector.

Fourier-transform infrared (FTIR) spectroscopy is a powerful technique for characterizing the optical properties of materials. It is widely used for measuring the vibrational motions of molecules, lattice vibrations in solids or other excitations in matter. FTIR allows one to get the full mid-infrared (MIR) spectrum of a sample (either in reflection or transmission) with a high spectral resolution. In this technique, light from a broadband blackbody source is driven through a Michelson interferometer and then focused on the sample. The reflected or transmitted signal is directed to a detector. The signal as a function of the reference mirror position (interferogram) is Fourier-transformed, giving us the full MIR spectrum of the sample in the frequency domain without the need of changing the wavelength. Detailed description of FTIR can be found elsewhere[3]. Figure S2 sketches a typical FTIR system.

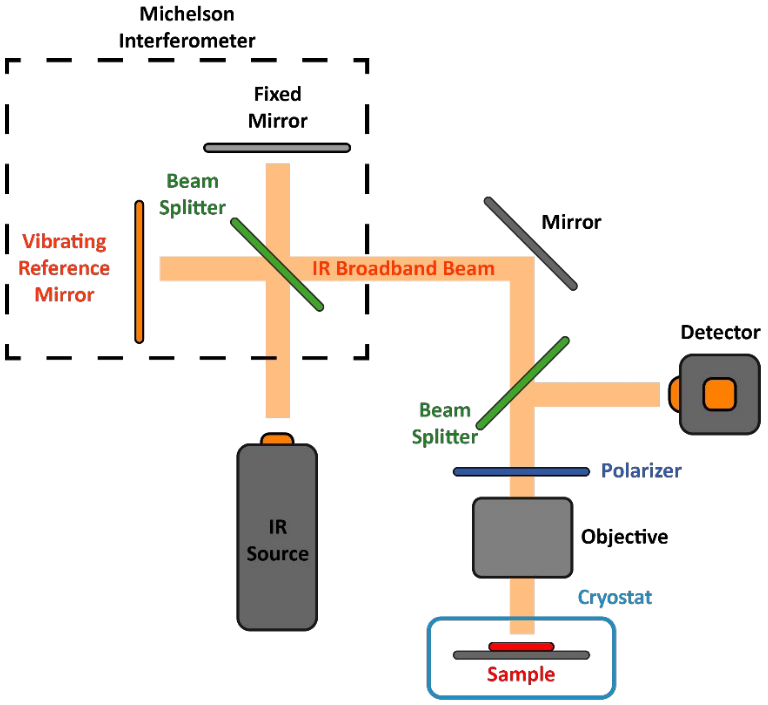

Figure S2. Schematic of a FTIR spectrometer in reflectance mode. Light from an IR source is driven to a Michelson interferometer (the optical path is divided into two branches, a fixed one and a movable one, creating a path difference between them). The beams from the two branches of the interferometer interfere in the beam splitter and are brought through a microscope (sketched here with an objective). The reflected beam is collected at a detector using another beam splitter.

Due to a finite numerical aperture of the focusing objective in the FTIR microscope the incident light is not completely perpendicular to the sample, forming its wavevector a small angle (up to 15° in our case) with respect to the vertical direction. As discussed below, this gives rise to certain spectral structures corresponding to the polarization orthogonal to the sample surface to be seen in the reflectivity spectra.

S2. Calculated Spectra of the Dielectric Function and Reflectivity for the Normal Angle of Incidence.

Calculated spectra of the dielectric function $\varepsilon_\alpha(\omega)$ of α-MoO_3 for the principal polarizations $\alpha =$ [100], [010], [001] are shown in Figure S3. We compute the theoretical reflectivity using the Fresnel formula for normal incidence:

$$R_\alpha(\omega) = |r_{p,\alpha}|^2 = \left|\frac{\sqrt{\varepsilon_\alpha(\omega)}-1}{\sqrt{\varepsilon_\alpha(\omega)}+1}\right|^2 \tag{S1}$$

For each polarization, one strong *Reststrahlen* band is observed as well as some weaker phonon features. As is seen in Figure 1 of the main text, the experimental and calculated RBs match very well.

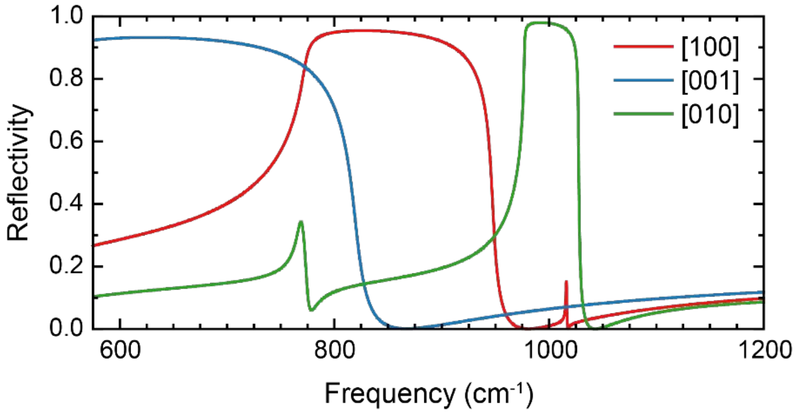

Figure S3. *ab initio* extracted reflectivity curves for α-MoO_3. Reflectivity curves for α-MoO_3 computed employing the *ab initio* calculated phonon parameters for the polarizations along the [100] (red), [001] (blue) and [010] (green) at 300 K. [100] and [001] are the in-plane directions and [010] is the out-of-plane direction. We can observe a high reflectivity band for each polarization, which are attributed to the presence of a *Reststrahlen* Band.

S3. Effect of the Finite Angle of Incidence on the Experimental Reflectivity Spectra.

Figure S4 presents a more detailed comparison between the experiment and theory for the polarizations [100] and [001]. One can see that both experimental spectra (red curves) show a small additional structure at around 1000-1010 cm^{-1}. Although it seems to match well the extra peak at 1015 cm^{-1}, found in the theoretical curve for the [100] polarizations (red curve in Figure S3 and light blue curve in Figure S4A and marked in the latter with a black vertical arrow), the actual situation is more subtle.

One problem with this straightforward interpretation is that this extra structure is not seen in the theoretical curve for the [001] polarization, contrary to the experiment. Second, if we calculate theoretical reflectivity for the angle of incidence of 15°, which matches specifications of the FTIR objective (dark-yellow curves in Figure S4A and S4B), a new structure appears at 1030 cm^{-1} for both [100] and [001] polarizations, as shown by dark-yellow arrows. As the structure is absent in the normal-incident spectra, it can only originate from the longitudinal optic (LO) mode along the [010] direction (as can be verified from the green curve in Figure S3). Another notable structure is a shoulder at about 800 cm^{-1} found in the experimental reflectivity for the [001] polarization. Such a feature is also formed in the calculated spectrum for the finite angle of incidence (see dark yellow curve in Figure S4B and indicated with a dark yellow arrow). Indeed, as shown in Figure S3 (green curve), the presence of this shoulder also matches the phonon mode along the [010] direction and therefore we can relate its presence to the oblique incidence of light.

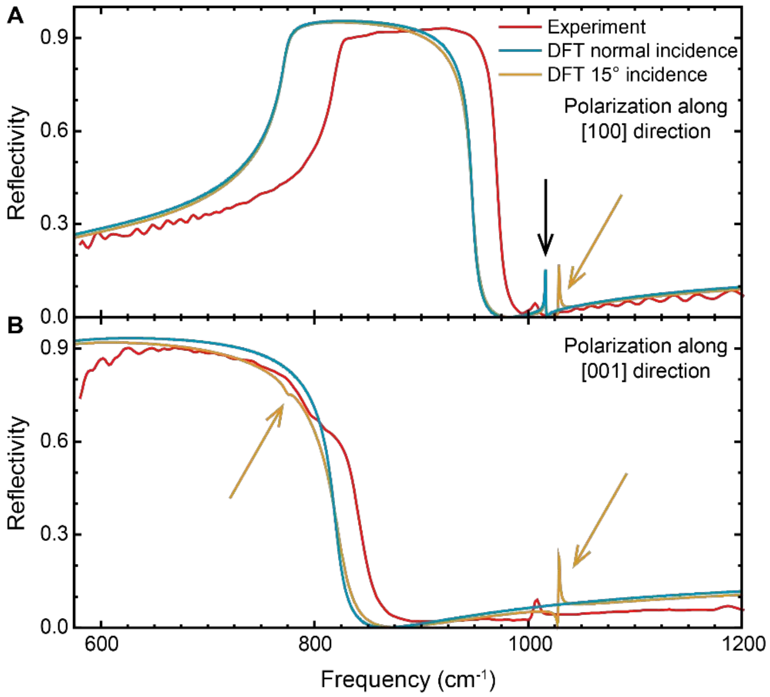

Figure S4. Comparison between the experimentally measured reflectivity curves and *ab initio* computed for normal incidence and 15° incidence at 300 K. Reflectivity curves for an α-MoO_3 stone measured (red curve) and computed by means of the phonon parameters obtained through *ab initio* calculations for normal (blue curve) and 15° incidence (orange curve) for light polarized along the [100] (a) and [001] (b) axes at 300 K. There are some features present for the calculated 15° incidence that do not appear for the normal incidence curves and are related to the presence of phonon excitations along the [010] direction at such frequencies.

The following question then arises: why do we miss in the experimental spectra for the [100] polarization the 1015 cm^{-1} phonon mode predicted by theory? Figure S5 shows a zoom-in graph of the experimental reflectivity curves at the surrounding frequencies. A splitting of this structure in two can be observed at the lowest temperature. Therefore, a plausible explanation is that the [100] phonon is too weak and broad to be seen in the 300 K spectrum. However, it shows up more clearly at 5 K as the lower-frequency peak in the 1010-1020 cm^{-1} spectral structure. The higher frequency peak (which is well seen at both temperatures) is the aforementioned LO phonon along the [010] direction. This explanation fully reconciles the experimental and theoretical spectra.

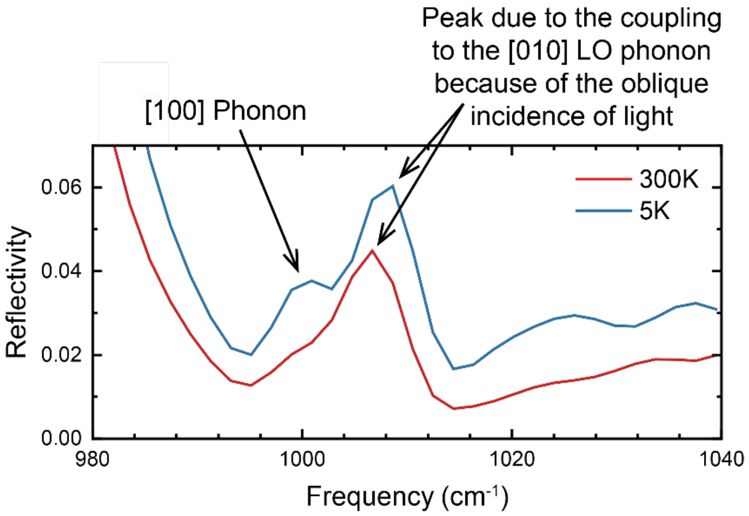

Figure S5. Reflectivity spectrum for α-MoO_3 polarized along the [100] with oblique incidence. Due to the broadening of the phonon peaks at high temperatures, the small oscillator strength and the frequency overlapping with the [010] LO phonon due to the oblique incidence, the phonon mode along the [100] direction at about 1000 cm^{-1} is only perceptible at low temperature.

S4. Polaritonic Wavelength, Group Velocity and Propagation Length: Experiment vs. Theory

In this section, we compare the experimentally obtained and theoretically calculated PhP wavelengths, λ_p, group velocities, v_g, and propagation lengths, L_p. Figure S6 shows the experimentally extracted PhP wavelength, $\lambda_p = 2\pi/k_p$ (panels A and B), and group velocity, $v_g = d\omega_0/dk_p$ (panels C and D), as a function of the excitation frequency and temperature for the lower (hyperbolic, A and C) and upper (elliptic, B and D, along the [001] direction) *Reststrahlen* Bands (L-RB and U-RB). λ_p was directly obtained by fitting the near-field profiles (as those shown in Figure 3B of the main text) with Equation 2 of the main text. On the other hand, v_g was calculated by fitting the PhP dispersion to a power function and performing its numerical derivative.

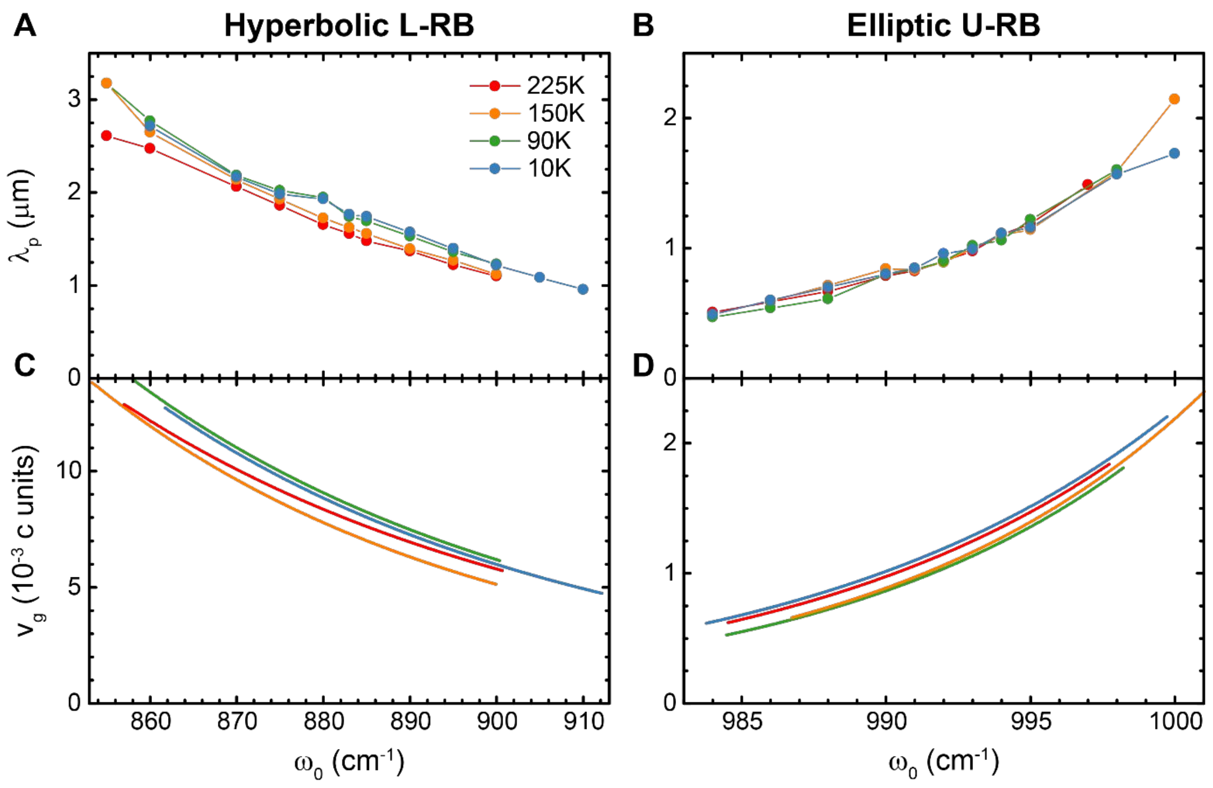

Figure S6. Experimental temperature dependence of the wavelength and group velocity of PhPs in α-MoO_3. a), b) PhPs wavelength in α-MoO_3 as a function of the incident frequency for temperatures from 10 K to 225 K within the hyperbolic (a) and elliptic (b) RBs. c), d) PhPs group velocity in α-MoO_3 as a function of the illuminating frequency for the hyperbolic (c) and elliptic (d) RBs for temperatures from 10 K to 225 K. Panels (b) and (d) refer to the α-MoO_3 [001] direction, similar results are expected for the [100] direction.

Regarding λ_p, we observe an increase/decrease as a function of frequency for the elliptic/hyperbolic RB, indicating a negative/positive phase velocity, in agreement with previous reports[4,5]. In terms of temperature dependence, we observe a different behavior in the two RBs: while a modest increase (around 5% in average in the selected frequency range) is observed in the hyperbolic regime when decreasing the temperature from 225 K to 10 K (Figure S6A), no changes are discernible in the elliptic regime (Figure S6B). With respect to v_g, we observe small values at all temperatures in both RBs (Figure S6C for the L-RB and Figure S6D for the U-RB), also in consistency with former results[4]. For example, in the L-RB we calculate a group velocity of $v_g = 1.9 \cdot 10^6$ m s^{-1}, corresponding to $6.5 \cdot 10^{-3}\ c$ with c the speed of light, at $\omega_0 =$ 895 cm^{-1} and $T =$ 10 K. In the U-RB, we obtain values of $2.5 \cdot 10^5$ m s^{-1} $= 8.6 \cdot 10^4\ c$ at $\omega_0 =$ 988 cm^{-1} and 10 K. Due to the variation of the polariton wavelengths in the hyperbolic

band (L-RB) as a function of temperature (Figure S6A), the group velocities at different temperatures in this RB are also different, with higher values at lower temperatures (Figure S6C). The group velocities in the elliptic regime are almost invariant with respect to temperature (Figure S6D).

Theoretically, PhPs wavelengths and group velocities can be calculated using the theoretical dispersion relation for electromagnetic modes in biaxial slabs embedded between two isotropic media[6]:

$$k_p = \frac{\rho}{d}\left[\tan^{-1}\left(\frac{\varepsilon_1 \rho}{\varepsilon_z}\right) + \tan^{-1}\left(\frac{\varepsilon_3 \rho}{\varepsilon_z}\right) + \pi l\right]; l \in \mathbb{Z} \quad \text{(S1)}$$

where k_p is the in-plane PhPs wavenumber, d is the α-MoO_3 flake thickness, ε_1 and ε_3 are the permittivities of the superstrate (air) and substrate (SiO_2), respectively, $\rho = i\sqrt{\varepsilon_z/(\varepsilon_x \cos^2\beta + \varepsilon_y \sin^2\beta\ )}$ with ε_x, ε_y and ε_z the material permittivity and β the angle between the [100] axis and the in-plane component of the wavevector. In particular, the PhPs wavelength is calculated as $\lambda_p = 2\pi/\Re(k_p)$ while the group velocity is extracted by performing the numerical derivative of $\omega_0(k_p)$. The calculated λ_p and v_g are shown in Figure S7 for temperatures ranging from 300 K (red curve) to 10 K (blue curve). They show a good qualitative agreement with the experimental results shown in Figure S6. We note that in analogy to the results shown in Figure 2 of the main text, the theoretical values of λ_p and v_g (as a difference to their trends/slopes) are not directly comparable to the experimental results due to the intrinsic frequency shifts obtained in DFT calculations.

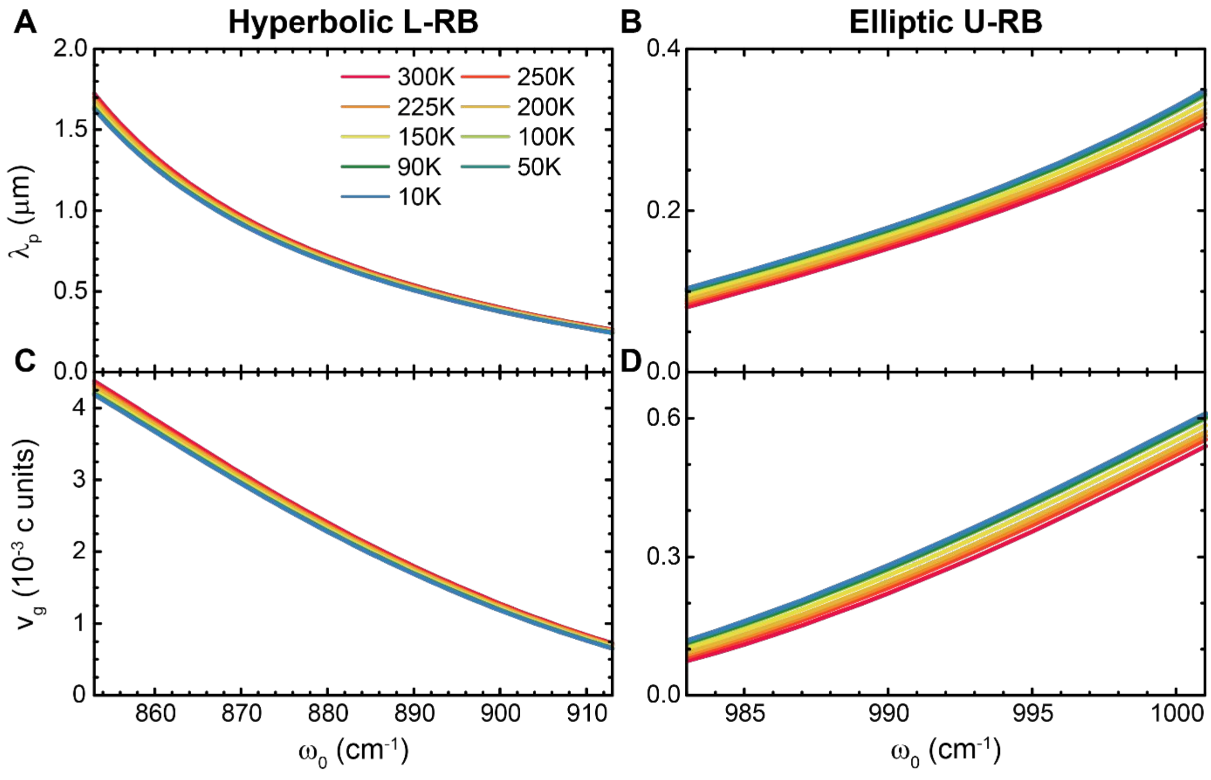

Figure S7. Theoretical temperature dependence of the wavelength and group velocity of PhPs in α-MoO_3. a), b) PhPs wavelength in α-MoO_3 as a function of the incident frequency for temperatures ranging from 10K to 300K for the hyperbolic (a) and elliptic (b) RBs. c), d) PhPs group velocity in α-MoO_3 as a function of the incident frequency for temperatures ranging from 10 K to 300 K for the hyperbolic (c) and elliptic (d) RBs. Ultra-slow group velocities are found in both regimes. Panels (b) and (d) refer to the α-MoO_3 [001] direction, similar results are expected for the [100] direction.

We will focus now on the PhPs propagation length as a function of temperature as the information about the damping mechanisms is encoded into it. The experimental propagation lengths are extracted by fitting s-SNOM near-field profiles (see Figure 3 of the main text) with Equation 2 of the main text. The theoretical values are obtained by taking the inverse of the imaginary part of the PhPs wavenumber ($1/\Im(k_p)$) given by Equation S1. Figure S8 plots both the experimental (Figure S8A for the L-RB and Figure S8B for the U-RB) and theoretical (Figure S8C for the L-RB and Figure S8D for the U-RB) results. In all cases, we observe the longest propagation length at the lowest temperature. However, in terms of frequency dependence, the longest propagation lengths are obtained in the proximity of ω_{TO} for the hyperbolic regime and in the proximity of ω_{LO} for the elliptic regime (due to its negative phase velocity). Both experimentally and theoretically, we find an average enhancement in the PhPs

propagation length when decreasing the temperature. In the experiment, this enhancement is about 30% in the L-RB and about 50% in the U-RB when decreasing the temperature from 225 K to 10 K. Table S1 shows a detailed comparison of the enhancement of the propagation length when decreasing the temperature from 225 K to 10 K ($\Delta L_p(\%) = 100 \cdot [L_p(10\text{ K}) - L_p(225\text{ K})]/L_p(225\text{ K})$) for two selected frequencies in each RB. The same general trend is theoretically obtained, showing the enhancement PhPs propagation length almost coinciding with the experimental ones at $\omega_0 =$ 900 cm^{-1} and $\omega_0 =$ 995 cm^{-1} (at $\omega_0 =$ 986 cm^{-1} there is a more significant difference). Taken together, these results are clear indications of an effective decrease of the PhPs damping channels with cooling down.

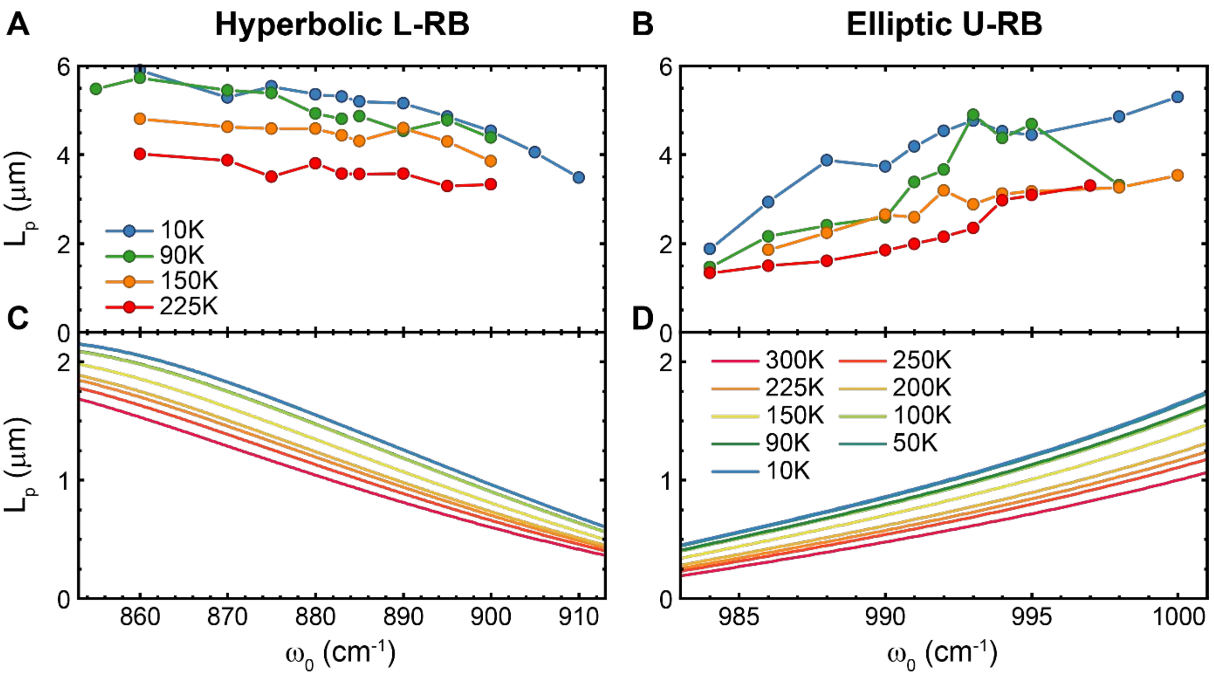

Figure S8. Temperature dependence of the propagation length of PhPs in α-MoO_3. PhPs propagation length in α-MoO_3 as a function of the incident frequency for temperatures ranging from 10 K to 300 K for the L-RB (panels a) and c)) and U-RB (panels b) and d)). (a) and (b) present the experimental values while (c) and (d) show the theoretically calculated results. The experimental values are obtained by fitting s-SNOM profiles (see Figure 3 of the main text) with Equation 2 of the main text, while theoretical values are calculated by taking the inverse of the imaginary part of the polariton wavenumber given by Equation S1. In all cases, the propagation length increases with cooling down. Panels (b) and (d) refer to the α-MoO_3 [001] direction, similar results are expected for the [100] direction.

ω_0 (cm^{-1})	$\Delta L_{p,\mathrm{exp}}$(%)	$\Delta L_{p,\mathrm{th}}$(%)
880	41	30
900	36	37
986	96	60
995	44	44

Table S4. Increase of the propagation length of PhPs in α-MoO_3 at low temperatures. Increase of the propagation length as a function of temperature, defined as: $\Delta L_p(\%) = 100 \cdot [L_p(10\text{ K}) - L_p(225\text{ K})]/L_p(225\text{ K})$ for some selected frequencies in each RB.

S5. Temperature-Dependent Study of the Polaritonic Lifetime in the Elliptical Band.

Although the presence of the inherent mismatch for the phonon positions does not allow us a direct comparison between polaritonic wavelengths, group velocities and propagation lengths between experiment and theory, polaritonic lifetime is a figure of merit that is almost constant within the whole RB except at the extremities. Figure S9 shows the *ab initio* calculated polaritonic lifetime at 300 K for α-MoO_3 in the hyperbolic regime with the parameters extracted in Section S4 of this Supplementary Information.

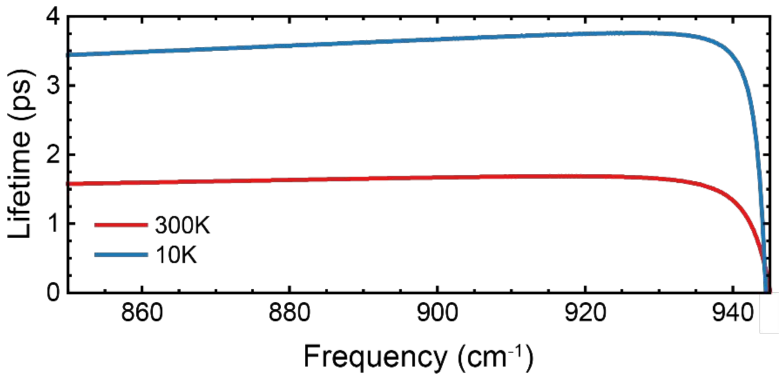

Figure S9. Frequency dependence of the PhP lifetime in α-MoO_3. PhP lifetime in the hyperbolic L-RB calculated using the permittivity of α-MoO_3 extracted from *ab initio* calculations. The flake thickness was set to 100 nm and BaF_2 was employed as substrate.

One can notice the almost flat-dispersive curve until frequencies close to the LO phonon. Therefore, as first approximation, we can directly compare the *ab initio* results with the experimental results at certain frequencies if, and only if, those frequencies are far from both

TO and LO phonon frequencies, as the ones selected for Figure 4 of the main text ($\omega_0 =$ 860 cm^{-1} and $\omega_0 =$ 895 cm^{-1}).

We adopted the same approximation for calculating the polaritonic lifetimes in the elliptical regime. Figure S10 presents the results of the phonon-polaritonic lifetimes calculated in the same way as the results presented in Figure 4 of the main text, employing the propagation lengths and group velocities presented in Supplementary Section S4 for the elliptical regime along the [001] crystallographic direction. One can notice a good agreement between experimental and theoretical results.

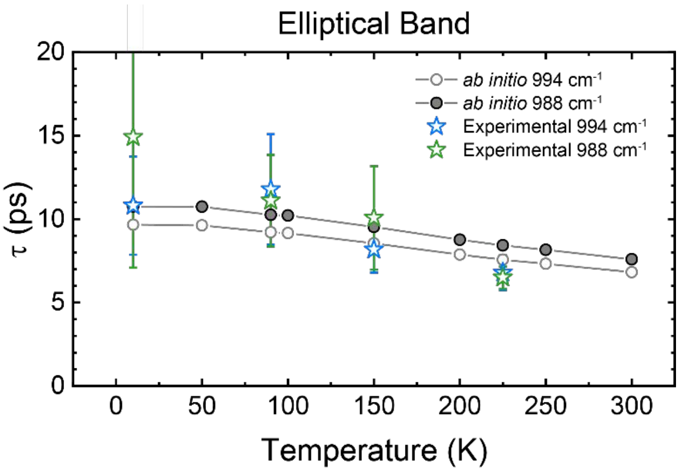

Figure S10. Temperature dependence of PhPs lifetimes in α-MoO_3 in the elliptical band along the [001] crystallographic direction. Theoretical (circles) and experimental (star symbols) PhPs lifetimes for a 104 nm-thick α-MoO_3 flake as a function of temperature for the elliptic *Reststrahlen* Band ($\omega_0 =$ 988 cm^{-1} and $\omega_0 =$ 994 cm^{-1}). Gray lines are guides to the eye.

For comparing numbers, at 10 K theoretical lifetimes reach values of $\tau_{ab\ initio} =$ 10.8 ps and $\tau_{ab\ initio} =$ 9.7 ps at $\omega_0 =$ 988 cm^{-1} and $\omega_0 =$ 944 cm^{-1}, respectively, whereas experimental values reach $\tau_{exp} =$ 15 ± 8 ps and $\tau_{exp} =$ 10 ± 3 ps at those frequencies, confirming that the third-order anharmonic phonon scattering processes are the responsible of the damping mechanisms of PhPs in α-MoO_3, as discussed in the main text.

Note that due to the inverted polaritonic dispersions in the U- and L-RB due to the different sign of the phase velocity, the longest lifetimes are found in the vicinity of ω_{TO} for the elliptic regime and in the vicinity of ω_{LO} for the hyperbolic regime.

References

[1] X. Chen et al., “Modern Scattering-Type Scanning Near-Field Optical Microscopy for Advanced Material Research,” Advanced Materials, vol. 31, no. 24, p. 1804774, Jun. 2019, doi: 10.1002/ADMA.201804774.

[2] N. Ocelic, A. Huber, and R. Hillenbrand, “Pseudoheterodyne detection for background-free near-field spectroscopy,” Appl Phys Lett, vol. 89, no. 10, p. 101124, Sep. 2006, doi: 10.1063/1.2348781/327139.

[3] P. R. Griffiths and J. A. De Haseth, “Fourier Transform Infrared Spectrometry: Second Edition,” Fourier Transform Infrared Spectrometry: Second Edition, pp. 1–529, Jun. 2006, doi: 10.1002/047010631X.

[4] W. Ma et al., “In-plane anisotropic and ultra-low-loss polaritons in a natural van der Waals crystal,” Nature, vol. 562, no. 7728, pp. 557–562, Oct. 2018, doi: 10.1038/s41586-018-0618-9.

[5] J. Taboada-Gutiérrez et al., “Broad spectral tuning of ultra-low-loss polaritons in a van der Waals crystal by intercalation,” Nat. Mater., vol. 19, no. 9, pp. 964–968, Sep. 2020, doi: 10.1038/s41563-020-0665-0.

[6] G. Álvarez-Pérez, K. V. Voronin, V. S. Volkov, P. Alonso-González, and A. Y. Nikitin, “Analytical approximations for the dispersion of electromagnetic modes in slabs of biaxial crystals,” Phys. Rev. B, vol. 100, no. 23, p. 235408, Dec. 2019, doi: 10.1103/physrevb.100.235408.
